# Symptomatic Patients without Epidemiological Indicators of HIV Have a High Risk of Missed Diagnosis: A Multi-Centre Cross Sectional Study

**DOI:** 10.1371/journal.pone.0162503

**Published:** 2016-09-07

**Authors:** Johanna Brännström, Veronica Svedhem, Gaetano Marrone, Örjan Andersson, Farshad Azimi, Anders Blaxhult, Anders Sönnerborg

**Affiliations:** 1 Unit of Infectious Diseases, Department of Medicine, Karolinska Institute, Karolinska University Hospital Huddinge, Stockholm, Sweden; 2 Department of Infectious Diseases, Karolinska University Hospital Huddinge, Stockholm, Sweden; 3 Department of Infectious Diseases, Sundsvall County Hospital, Sundsvall, Sweden; 4 Department of Infectious Diseases, Gävle County Hospital, Gävle, Sweden; 5 Department of Clinical Science and Education, Venhälsan, Stockholm South General Hospital, Stockholm, Sweden; 6 Division of Clinical Microbiology, Department of Laboratory Medicine, Karolinska Institute, Stockholm, Sweden; National and Kapodistrian University of Athens, GREECE

## Abstract

**Objectives:**

One quarter of HIV-1 positive individuals in Sweden present for care with HIV or AIDS associated conditions without an HIV test (missed presentations) and 16% report neglect of such symptoms. The objective of this study was to identify risk factors for these missed opportunities of HIV-1 diagnosis.

**Methods:**

A national study, recruiting 409 newly diagnosed HIV-1 infected adults over a 2.5-year period, was performed. Logistic regression models tested the relationship between missed presentation and patient’s neglect versus socio-demographic and behavioural risk factors. Additionally the initiator of the HIV test was assessed.

**Results:**

The odds for a missed presentation was lower for migrants (from East Europe, Asia, and Pacific (East): OR 0.4 (0.2–0.8); Sub-Saharan Africa (SSA): 0.3 (0.2–0.6); other: 0.5 (0.2–1.0)), compared to patients born in Sweden, just as symptoms neglected by the patient (East (0.3 (0.1–1.0); SSA (0.4 (0.2–0.8)). The latter was also lower for men who have sex with men (0.5 (0.2–1.0)), compared to patients infected heterosexually. Patients infected in the East, with present/previous substance use or a previous negative HIV test were more likely to take the initiative to test on their own, whereas those >50 years and with a previously missed presentation had significantly reduced odds, p<0.05.

**Conclusions:**

Individuals without epidemiological indicators of HIV are more likely to have a history of missed presentations, to neglect symptoms and are less prone to take an initiative to test for HIV themselves. It is important to further implement testing to include all patients with symptoms and conditions indicative of HIV.

## Introduction

The majority of patients with human immunodeficiency virus type 1 (HIV-1) infection are diagnosed at a late stage of infection [1, 2], a condition associated with increased morbidity, mortality [3], higher health care costs [4] and risk of onward transmission [5]. E.g. in Sweden, 58% of the patients diagnosed in 2009 to 2012 [6] were late presenters (LP), defined as <350 CD4+ T-cells/mm^3^ or AIDS at diagnosis [2]. Although HIV-1 infected patients are most often asymptomatic for several years [7], a considerable number of patients seek health care with conditions indicative of HIV, so called indicator diseases [8], without being offered an HIV-test. The predominant strategy of risk factor based HIV testing, used in most European countries, thus fails to detect a substantial part of the HIV infected. One explanation is that many individuals do not consider themselves at risk and the health care providers also fail in risk assessments [9].

It has previously been shown that one quarter of newly HIV-1 diagnosed patients in Sweden present for care with AIDS- or HIV-associated conditions during the three years before diagnosis, without being HIV tested. Additionally, 16% of patients report HIV associated symptoms that did not cause them to seek health care [6].

The aim of the present study was therefore to assess these missed opportunities to diagnosis; to identify the characteristics of the patients most likely to be missed at seeking health care and the ones most likely to neglect their symptoms. In addition we also assessed the initiator of the diagnosing HIV test to characterize the patients most likely to take the initiative to test themselves.

## Material and Methods

### Study setting and study population

Eligible for the study were all patients (n = 575), ≥18 years old, who were newly diagnosed (within six months) with HIV-1 from the 1^st^ of October 2009 to the 31^st^ of January 2012, at twelve clinics in Sweden. This cohort represented three quarters of all newly diagnosed patients from all parts of the country with an equal geographical distribution including the major cities and county clinics and has previously been described in detail [6]. All participants provided their written informed consent and the study was approved by the Regional Ethical Review Board in Stockholm, Sweden (2009/1029-31/1-4).

### Data collection and definition of risk factors

Demographic data (gender, age, country of origin, estimated country and route of transmission), CD4+ T-cell counts, date of first positive HIV serology, and any AIDS diagnosis were collected from the national InfCare HIV database. For those who agreed to participate, the physician completed a questionnaire, based on the medical history obtained from the patient and hospital records. The questionnaire collected information from the previous three years before diagnosis about: i) missed AIDS conditions at presenting for care [10]; ii) predefined missed HIV associated conditions at presenting for care; iii) any HIV or AIDS symptoms neglected by the patient. Information was also collected about: iv) psychiatric illness; v) present or previous drug abuse; vi) immigration date; vii) any previous negative HIV test in Sweden; viii) initiator of the HIV test and reason for testing (patient´s initiative or physician´s initiative due to symptoms or screening). Symptoms reported when the patients were documented to be HIV-negative were excluded.

The patients were classified as non-late presenters (nLP) or late presenters (LP): CD4+ T-cell count < 350 cells/mm^3^ and/or AIDS (within 3 months of diagnosis) [2]. Patients with primary HIV infection (PHI) were defined as non-LP, irrespective of the CD4+ T-cell count [6].

The countries of origin and transmission were grouped into regions based on the Joint United Nations Programme on HIV/AIDS (UNAIDS) [11] classification and further grouped into Sweden, East (East Europe/Asia and the Pacific), SSA (Sub-Saharan Africa), Other (Western Europe, The Americas, North Africa, Israel, and the Middle East) and Unknown, for inferential statistics, in order to have a lower number of categories and more statistical power.

### Outcome definitions

Primary outcomes were two types of missed opportunities to HIV test during the three years previous to the diagnosis: 1) “Missed presentation”, defined as a missed HIV diagnosis at presenting for care with a clinical indicator for HIV testing; 2) “Patients neglect”, defined as the presence of any HIV or AIDS associated symptoms, experienced by the patient, without seeking medical care at all or not until additional symptoms evolved.

Clinical indicators for HIV testing were all AIDS associated events [10] and the following HIV associated conditions, representing an extended version of the conditions used in the HIDES-1 study by the HIV in Europe [12]: sexually transmitted infections (STIs), hepatitis B/C, herpes zoster, cervical or anal dysplasia/cancer, seborrheic dermatitis or exanthema, unexplained anaemia, thrombocytopenia or neutropenia > 4 weeks, oral thrush without previous use of antibiotics (the last month), fever of unknown origin (FOU) >1 week, unexplained lymphadenitis, and/or unexplained elevation of the erythrocyte sedimentation rate (ESR). For clinical indicators requiring investigations (wasting, seborrheic dermatitis/exanthema, penia, FOU, lymphadenitis, SR-elevation), “missed presentation” was defined as HIV test not performed within 1 month. For the remaining AIDS defining conditions, other STIs, hepatitis, oral thrush without previous antibiotics and herpes zoster, the opportunity was considered missed if it had not triggered a test immediately.

Secondary outcome was the initiator of the HIV test, which was analysed by comparing the patient as the initiator of the test versus any other type of testing initiator (physician, screening, contact tracing, mother’s health or blood donation).

### Statistical analysis

Data was summarized with descriptive statistics (mean, median, standard deviation, percentiles for numerical variables, frequencies and percentages for categorical variables). Cross tabulations with Chi-Square or Fisher test were used to test for un-adjusted relationship between outcomes and categorical independent variables. For independent numerical variables t-test, Wilcoxon rank sum test and Kruskal Wallis test were used to compare mean and medians in two and more groups respectively, using Bonferroni correction for multiple comparisons for post-hoc tests.

A binary logistic regression model was used to identify significant predictors. For all variables a screening cross-tabulation was made to look for risk factors being of interest. Variables with P-value less then 0.2 and all the basic demographic variables were included in a backward (with 0.20 as significance level for removal from the model) stepwise logistic regression model. Crude and adjusted odds ratios (OR) with their 95% confidence intervals were presented. P-value <0.05 was considered significant in the final models.

Data analysis was performed using the STATA software 13 (Stata Corp. College Station, Texas, USA).

## Results

Altogether 409 (71%) patients completed the questionnaire and were included. Reasons for not participating were; rejection 11%; medical 7%, loss to follow up 6%, and logistic 5%.

### Patient demographics

The patient demographics are depicted in Table 1. In brief, two thirds were male and the mean age at diagnosis was 40 years. The majority were infected heterosexually (53%) and one third (35%) were men who have sex with men (MSM). The country of origin was Sweden in 38% and Sub-Saharan Africa in 33%. Fifty-seven per cent (233/409) were LP with a median CD4+ T-cell count of 150/mm^3^ (mean 159, SD 111) at diagnosis, compared to 480 cells/mm^3^ (mean 540, SD 252) for the nLP. No significant differences were found between the patients who filled in the questionnaire (n = 409) and those who did not (n = 166) regarding gender, age, the main routes of transmission, HIV stage or CD4+ T-cell count at diagnosis (S1 Table).

**Table 1:** Descriptive and bivariate analysis of explanatory variables for “Missed presentation” and “Patients neglect” three years preceding HIV-1 diagnosis in 409 newly diagnosed patients. MSM, men who have sex with men; PWID, people with injecting drug use; SSA, sub-Saharan Africa; **‘**East**’**, Eastern Europe, Asia and the Pacific region; **‘**Other**’**, Western Europe, North and Latin America, the Caribbean, North Africa, Israel and the Middle East.

Characteristics		Missed presentation	Missed presentation	P-value	Patients Neglect	Patients Neglect	P-value
	Total	Yes	No		Yes	No	
	(Col %)	(Row %)	(Row %)		(Row %)	(Row %)	
***Total cohort***	409	112 (27.4)	297 (72.6)		65 (15.9)	344 (84.1)	
**Gender**				0.052			0.185
Female	136 (33.2)	29 (21.3)	107 (78.7)		17 (12.5)	119 (87.5)	
Male	273 (66.8)	83 (30.4)	190 (69.6)		48 (17.6)	225 (82.4)	
**Age (years)**				0.150			0.339
< = 30	88 (22.0)	18 (20.4)	70 (79.6)		13 (14.8)	75 (85.2)	
31–40	151 (36.9)	42 (27.8)	109 (72.2)		25 (16.6)	126 (83.4)	
41–50	96 (23.5)	25 (26.0)	71 (74.0)		11 (11.5)	85 (88.5)	
> 50	74 (18.1)	27 (36.5)	47 (63.5)		16 (21.6)	58 (78.4)	
Mean (SD)	40.1 (11.5)	42.4 (12.1)	39.2 (11.2)	**<0.05**	40.9 (12.5)	40.0 (11.3)	0.722
**Route of transmission**				0.095			0.816
Heterosexual	215 (52.6)	52 (24.2)	163 (75.8)		37 (17.2)	178 (82.8)	
MSM	142 (34.7)	50 (35.2)	92 (64.8)		22 (15.5)	120 (84.5)	
PWID	16 (3.9)	3 (18.8)	13 (81.2)		1 (6.2)	15 (93.8)	
Blood	6 (1.5)	2 (33.3)	4 (66.7)		1 (16.7)	5 (83.3)	
Unknown/Other	30 (7.3)	5 (16.7)	25 (83.3)		4 (13.3)	26 (86.7)	
**Country of origin**				**<0.001**			0.160
Sweden	156 (38.1)	61 (39.1)	95 (60.9)		29 (18.6)	127 (81.4)	
SSA	136 (33.3)	23 (16.9)	113 (83.1)		17 (12.5)	119 (87.5)	
East	58 (14.2)	12 (20.7)	46 (79.3)		6 (10.3)	52 (89.7)	
Other	55 (13.5)	14 (25.4)	41 (74.6)		13 (23.6)	42 (76.4)	
Unknown	4 (1.0)	2 (50.0)	2 (50.0)		0 (0.0)	4 (100.0)	
**Country of transmission**				**<0.05**			0.795
Sweden	154 (37.7)	56 (36.4)	98 (63.6)		26 (16.9)	128 (83.1)	
SSA	113 (27.6)	20 (17.7)	93 (82.3)		15 (13.3)	98 (86.7)	
East	71 (17.4)	17 (23.9)	54 (76.1)		10 (14.1)	61 (85.9)	
Other	47 (11.5)	11 (23.4)	36 (76.6)		9 (19.2)	38 (80.8)	
Unknown	24 (5.9)	8 (33.3)	16 (66.7)		5 (20.8)	19 (79.2)	
**Psychiatric illness**				0.343			0.424
No	364 (89.0)	267 (73.4)	97 (26.7)		308 (84.6)	56 (15.4)	
Yes	45 (11.0)	30 (66.7)	15 (33.3)		36 (80.0)	9 (20.0)	
**Drug use**				0.429			0.542
No	314 (76.8)	225 (71.7)	89 (28.3)		266 (84.7)	48 (15.3)	
Yes	95 (23.2)	72 (75.8)	23 (24.2)		78 (82.1)	17 (17.9)	
**Previous negative test**				0.298			0.664
No	211 (51.6)	160 (75.8)	51 (24.2)		178 (84.4)	33 (15.6)	
Yes	176 (43.0)	121 (68.8)	55 (31.2)		149 (84.7)	27 (15.3)	
Unknown	22 (5.4)	16 (72.7)	6 (27.3)		17 (77.3)	5 (22.7)	

### Missed opportunities

Of all patients (n = 409), 37% had at least one missed opportunity; 27% had a missed presentation, and 16% had neglected HIV and/or AIDS symptoms. The LP with missed presentations had a mean of 2.1 (SD +/- 1.2) missed presentations compared to 1.2 (SD +/- 0.4) among the nLPs (p<0.001). The probability of being missed was twice as high among LPAH compared to nLP (OR 2.0; 95% CI 1.2–3.1, p<0.01). The CD4+ T-cells at diagnosis was lower for those who had a missed presentation (150 versus 290 cells/mm^3^) (p = 0.0001) (data not shown).

#### Missed AIDS or HIV-associated presentations

**Fig 1:**
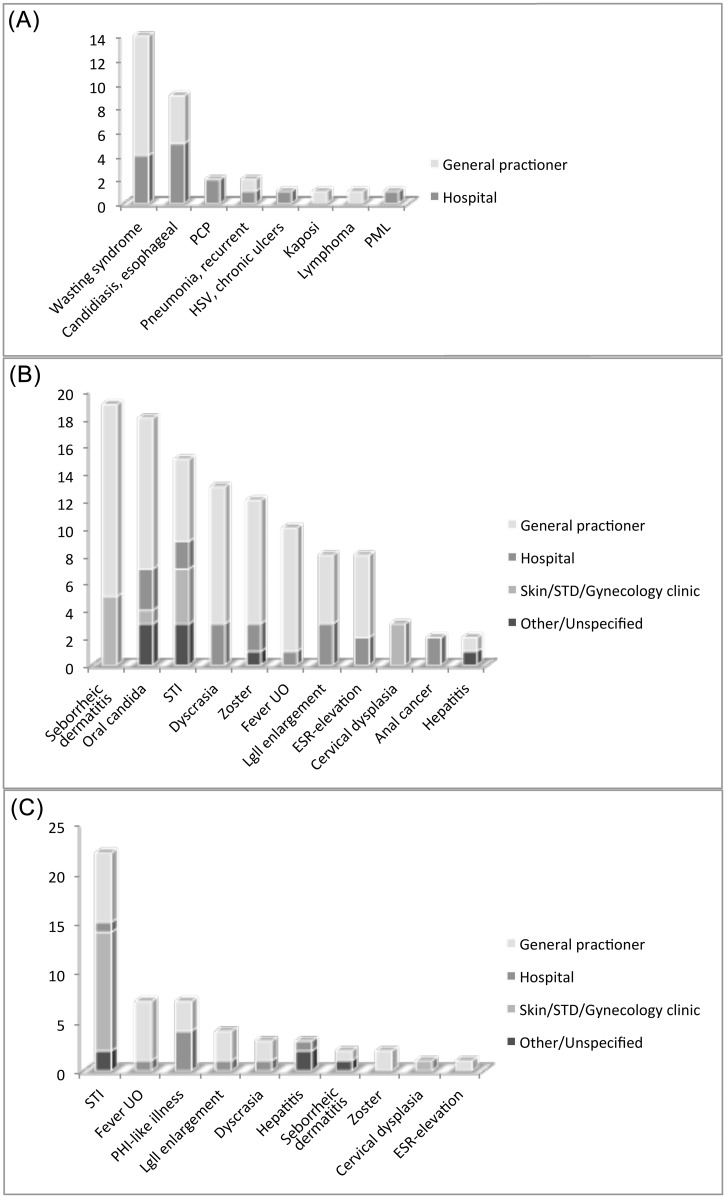
Missed HIV diagnosis at presenting for care with AIDS- and/or HIV associated conditions (n = 112, 27%), stratified by health care facility. A. 31 missed AIDS-associated presentations in 23 patients. B. 110 missed HIV-associated presentations in 60 late presenters. C. 52 missed HIV-associated presentations in 44 non-late presenters. PCP: Pneumocystis Pneumonia; HSV: Herpes Simplex Virus; PML: Progressive Multifocal Leukoencephalopathy; STI: Sexually Transmitted Infection; ESR: Erythrocyte Sedimentation Rate; PHI: Primary HIV Infection; Lgll: Lymphnodes.

Twenty-three (6%) patients (all LP) had presented for care, at least once, with one or more AIDS defining conditions (n = 31) without an HIV test, mostly in primary care (55%). Most common were wasting syndrome (n = 14) and Candida esophagitis (n = 9) (Fig 1A).

One-hundred four (25%) patients had presented for care, at least once, with one or more HIV associated conditions (n = 162). For the LP the most common conditions were seborrheic dermatitis (n = 19) and oral candida (n = 18). Among the nLP another STI (n = 22) and PHI-like illness, including also fever and lymph node enlargements, (n = 18) were the most frequent findings. Most missed HIV associated presentations were seen in primary care (58%), but several also at STI/skin clinics (16%) (Fig 1B and 1C).

**Fig 2:**
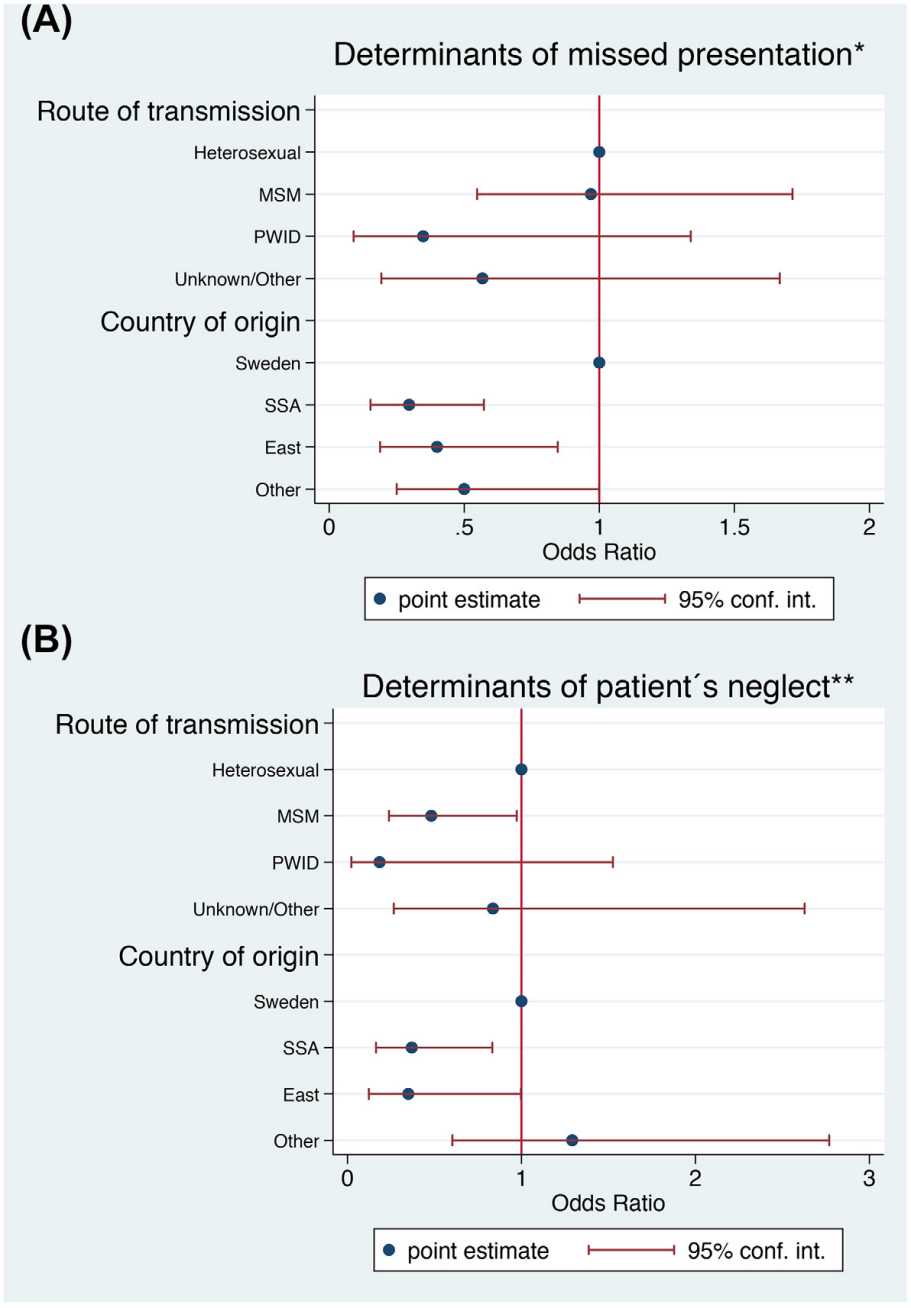
Multivariable logistic regression models for the determinants of “Missed presentation” and “Patient´s neglect” in 409 newly diagnosed patients. * The model was adjusted for: route of transmission. ** The model was adjusted for: age.

In a multivariable regression model, adjusted for route of transmission, patients from East or SSA had a reduced odds of missed presentations (OR 0.4; 95% CI: 0.2–0.8, p<0.05; OR 0.3; 95% CI: 0.2–0.6, p<0.001, respectively) compared to patients born in Sweden (Fig 2A).

#### Patient´s neglect

Sixty-five patients had neglected 92 HIV and/or AIDS symptoms three years previous to diagnosis. For LP, weight loss (19%) and fatigue (15%) were most often neglected. Among nLP a “PHI like syndrome” was most common, definitively described by 38% and possibly by another 35% (data not shown).

In a multivariable analysis (OR; 95% CI), adjusted for age, patients from SSA (0.4; 0.2–0.8) or East (0.3; 0.1–1.0) were less likely to neglect compared to those from Sweden. This was true also for MSM (0.5; 0.2–1.0) compared to heterosexuals (Fig 2B).

### Initiator of testing

The initiator of the HIV test was; patient: 25%; physician due to symptoms: 33%; screening: 41% (high prevalent groups: 23%; contact tracing: 11%; mothers health/blood donors: 7%); other/unknown: 2%. A difference between males and females was seen with regard to reason to test; own initiative: 30% vs 14%; physician due symptoms: 34% vs 29%; screening: 33% vs 56% (p<0.001). The proportion diagnosed by a physician due to symptoms increased with age; 55% among those >50 years compared to 27% among those <50 years (p<0.001). Among heterosexuals, 19% were tested on their own initiative compared to 39% among the MSM (p<0.001).

Among Swedish born patients, 39% were tested on physician initiative due to symptoms and 35% by their own initiative. Among patients from the East, the reason for testing was equally distributed whereas the majority (64%) of patients from SSA were tested due to screening and only 12% on their own initiative, which was significantly different from the other groups (p = 0.001).

**Fig 3:**
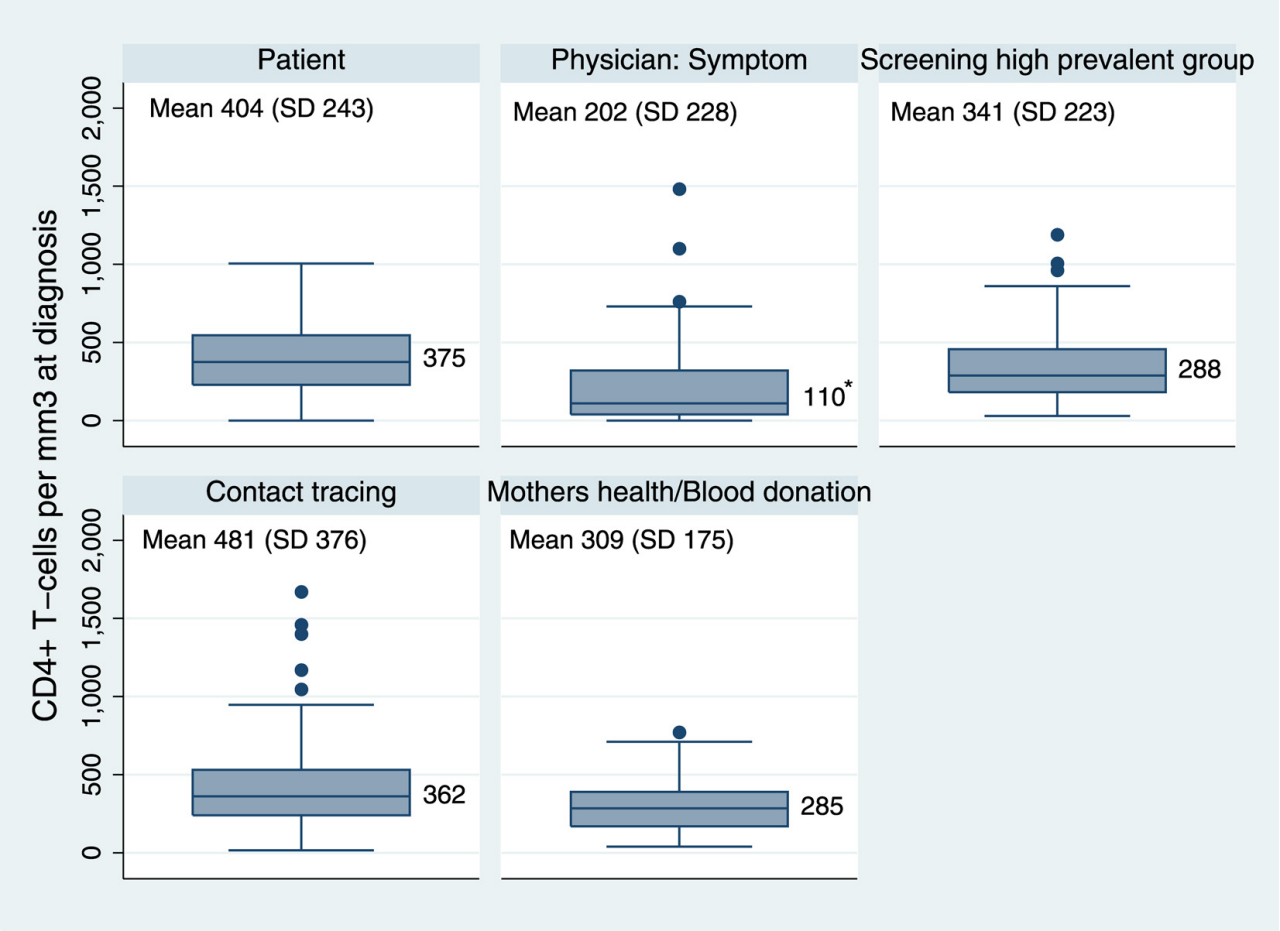
CD4+ T-cell count at diagnosis, depending on the initiator of/reason for HIV-testing. *The Kruskal Wallis test shows significant differences in median CD4 count between physician as initiator of the test and all other groups

Median CD4+ cells at diagnosis varied depending on mode of testing; own initiative: 375 cells/mm^3^; screening 300 cells/mm^3^; physician initiated due to symptoms: 110 cells/mm^3^ (Fig 3). The proportion of LP similarly increased from 38% among those with an own initiative to test to 54% among those tested due to screening and 74% among those tested due to symptoms (S2 Table).

#### Determinants of patient’s initiative

In a multivariable model, adjusted for gender and route of transmission, patients aged >50 years had a reduced odds ratio for testing on their own initiative (OR 0.4; 95% CI: 0.1–0.9) compared to those <30 years, just as those who had a history of previous health care contacts with a missed presentation (OR 0.5 (95% CI: 0.3–0.9) compared to those who did not. Patients infected in East Europe and Asia (OR 3.6; 95% CI: 1.6–8.2) or in countries defined as Other (OR 2.5; 95% CI: 1.1–5.7) had increased odds of having been tested on their own initiative compared to those infected in Sweden. Patients with a history of drug use (OR 1.8; 95% CI: 1.0–3.4) or a previous negative test (OR 2.9; 95% CI: 1.5–5.7) were also more prone to test on their own initiative (Table 2).

**Table 2:** Bivariate and multivariable logistic regression models for the patient as the initiator of the HIV-test in 409 newly diagnosed patients. CI, confidence interval; OR, odds ratio; MSM, men who have sex with men; PWID, people with injecting drug use; SSA, sub-Saharan Africa; **‘**East**’**, EasternEurope, Asia and the Pacific region; **‘**Other**’**, Western Europe, North and Latin America, the Caribbean, North Africa, Israel and the Middle East. Significant results are shown in bold.

	Crude OR	Adjusted OR	P-value
	(95% CI)	(95% CI)	
**Gender**			
Female	1	1	
Male	**2.7 (1.6–4.7)**	1.8 (0.9–3.8)	0.104
**Age**			
< = 30	1	1	
31–40	1.1 (0.6–2.0)	1.0 (0.5–2.0)	0.992
41–50	1.1 (0.5–2.1)	0.7 (0.3–1.6)	0.454
> 50	0.7 (0.3–1.5)	**0.4 (0.1–0.9)**	**<0.05**
**Route of transmission**			
Heterosexual	1	1	
MSM	**2.8 (1.7–4.5)**	1.3 (0.6–2.8)	0.466
PWID	1.4 (0.4–4.7)	0.5 (0.1–1.9)	0.309
Blood	-	-	-
Unknown/Other	0.5 (0.1–1.7)	0.3 (0.1–1.3)	0.122
**Country of origin**			
Sweden	1		
SSA	0.2 (0.1–0.5)		
East	0.9 (0.5–1.7)		
Other	0.6 (0.3–1.2)		
Unknown	-		
**Country of transmission**			
Sweden	1	1	
SSA	**0.2 (0.1–0.5)**	0.8 (0.3–2.1)	0.598
East	1.3 (0.7–2.4)	**3.6 (1.6–8.2)**	**<0.005**
Other	1.7 (0.9–3.5)	**2.5 (1.1–5.7)**	**<0.05**
Unknown	0.7 (0.2–1.9)	1.7 (0.5–5.7)	0.422
**Psychiatric illness**			
No	1		
Yes	1.1 (0.5–2.2)		
**Drug use**			
No	1	1	
Yes	**2.4 (1.4–3.9)**	**1.8 (1.0–3.4)**	**<0.05**
**Previous negative test**			
No	1	1	
Yes	**3.4 (2.1–5.4)**	**2.9 (1.5–5.7)**	**<0.005**
Unknown	0.3 (0.0–1.9)	0.2 (0.0–1.5)	0.119
**Missed Presentation**			
No	1	1	
Yes	0.6 (0.4–1.1)	**0.5 (0.3–0.9)**	**<0.05**

## Discussion

Indicator disease guided testing is one way forward to identify undiagnosed HIV infected patients [8]. The high relevance of this approach is confirmed in our study, in which 27% of the patients had presented for care, at least once, with HIV and/or AIDS-associated conditions without that an HIV test had been performed during three years before diagnosis. Additionally, 16% of the patients had neglected HIV associated symptoms during the same period [6].

Wasting syndrome was the most commonly missed AIDS condition, whereas oral candida, other STI, seborrheic dermatitis, PHI-like illness and blood dyscrasia dominated among the HIV associated conditions. The four latter were all evaluated in the HIDES-1 study, where a prevalence of HIV among those tested well exceeded the 0.1% needed to reach cost-effectiveness [12]. To educate clinicians about HIV and to incorporate the indicator guided testing, as proposed by the HIV in Europe initiative [13], further into clinical practice is essential, not the least among general practioners and STI/Skin clinics, where most of our patients were seen, but also missed.

Whereas missed opportunities to diagnose HIV infected patients seeking health care with indicator conditions is a well-known problem [9, 12, 14–17] there are, to our knowledge, limited reports on who do not get tested by the physician at presenting for care [9, 15–17] and no reports on who experience symptoms without seeking health care. The vast majority of LP in Sweden are migrants [6], but in the present study we showed that symptomatic patients from high prevalent countries were less likely to be missed by the physician at seeking health care and also to neglect their symptoms, compared to patients born in Sweden. The finding of a higher testing rate of migrants shows a good adherence to the epidemiological indicators for testing among health care professionals [17]. Somewhat surprising, there was no reduced risk in being missed for MSM. This could partly be explained by sexual orientation not being as obvious to the physician as origin from an epidemic area and seldom asked for. The observation is also in line with a French study, where half of the MSM actually clearly stated there sexual orientation, but were still not offered testing [9].

The reasons for physicians not offering a test are diverse, such as lack of training, self confidence in offering a test [18], concerns about the consent process, and competing priorities [19]. An HIV test is often primarily offered to patients perceived to be at high risk [20]. Additionally there are barriers for the symptomatic patient to seek health care, but we found that missed diagnosis at presentation by the health care provider was of greater importance than that of symptoms neglected by the patient.

Patients with a history of at least one missed diagnosis had a lower T-cell count (median 150 vs 290 cells/mm^3^) and had twice as often an advanced disease. A reduction in late presentation with as much as 50% in those tested after having presented with an indicator disease, compared to those not tested, have been reported [16]. With as many as 25% of the nLPs reporting missed presentations it is evident that great improvements can be made.

We showed an evident advantage of the existing screening programmes and also if the patient was tested by her/his own initiative, resulting in a good chance of an early diagnosis. However, with the present routines of health examination at immigration being inadequate, as shown in our recent study [6], and overall few patients with an own initiative to test, improvements are warranted.

The patients infected abroad or with a history of drug use took initiative to a test more frequently. Similarly, patients (mainly MSM) with a history of a previous negative test asked for a new test more often than those not tested before. This could reflect a higher perceived risk but also a greater availability of HIV screening, like STI clinics and drug addiction treatment centres [21]. On the contrary, patients >50 years were tested less often on their own initiative, which could partly explain the high proportion of LP among older individuals [6, 22–28]. Also here a low perception of risk has been proposed as a major explanation [15, 29]. To increase awareness of HIV and the availability of HIV testing sites for the general population, including the elderly, is both justified and necessary.

In summary we can establish that even though many migrants have missed opportunities of HIV testing at immigration the “HIV awareness”, both within health care and among individuals, are higher for those belonging to the classical epidemiological risk-groups, not the least when symptoms evolve, demonstrated by the lower patient´s neglect of symptoms in these group. However, whereas patients with a “risk-behavior” have a high probability to initiate a test themselves, often at an early stage, many migrants do not initiate the test, but seek healthcare, when becoming ill.

In our study, with consecutively recruited patients, all had a documented CD4+ T-cell count allowing us to calculate true median values at diagnosis and to classify all patients according to LP-status. The data collection was equally based on the medical history and hospital journals allowing for a good accuracy of the data. However, some limitations can be discussed. Considering that we only assessed a specified number of HIV associated conditions and, in order to minimize recall bias, restricted the patient history to three years, our figures are likely to be an underestimation. An expanded timeframe would most likely further emphasize our results and the extent of the problem. The last patient was included in year 2012. However, in view of that the HIV epidemic has not changed its face with regard to e.g. demographics of newly diagnosed patients, national guidelines for testing or the structure of the HIV health care in Sweden, we do believe that our data still is representative for the current situation in 2016. For the patient as the HIV test initiator it is possible that among the migrants tested soon after arrival in Sweden, several could have been tested on their own initiative if not having been offered the test. In a sensitivity analysis excluding all patients diagnosed within two months, when the health examination of migrants is recommended to be performed, however gave similar results as the one reported. Nor did this exclusion of patients change the results regarding missed diagnosis at presentation or neglect.

In conclusion, the study implies that in the best-case scenario 37 percent of the patients in our setting could have been diagnosed at an earlier stage as a result of identifications of symptoms alone. Strategies to identify these patients, without obvious epidemiological indicators of HIV, and other undiagnosed individuals should have the highest priority in the combat against the HIV-1 epidemic. Adding to this the previously described missed opportunity to test at immigration, seen in as many as two thirds of migrants [6] there is much to gain by improved implementation of testing strategies.

## Supporting Information

S1 Table Comparison of basic demographics for the patients participating in the study and not. MSM, men who have sex with men; PWID, people with injecting drug use; SSA, sub-Saharan Africa; **‘**East**’**, Eastern Europe, Asia and the Pacific region; **‘**Other**’**, Western Europe, North and Latin America, the Caribbean, North Africa, Israel and the Middle East.
(DOCX)

S2 Table Descriptive and bivariate analysis of explanatory variables for the initiator of/reason for HIV-testing. MSM, men who have sex with men; PWID, people with injecting drug use; SSA, sub-Saharan Africa; **‘**East**’**, Eastern Europe, Asia and the Pacific region; **‘**Other**’**, Western Europe, North and Latin America, the Caribbean, North Africa, Israel and the Middle East.
(DOCX)
