# Supplementary material for: Symptomatic Patients without Epidemiological Indicators of HIV Have a High Risk of Missed Diagnosis: A Multi-Centre Cross Sectional Study
Source: PLoS One. 2016 Sep 7;11(9):e0162503. doi: 10.1371/journal.pone.0162503 (PMC5014346; doi:10.1371/journal.pone.0162503)
Supplement: S1 Table — MSM, men who have sex with men; PWID, people with injecting drug use; SSA, sub-Saharan Africa; ‘East’, Eastern Europe, Asia and the Pacific region; ‘Other’, Western Europe, North and Latin America, the Caribbean, North Africa, Israel and the Middle East. (DOCX) [file pone.0162503.s001.docx]

**S1 Table.** **Comparison of the basic demographics for the patients participating in the study and not.**

| **Characteristics** | **Total**  **(Col %)** | **Included**  **(Row %)** | **Not Included**  **(Row %)** | **P-value** |
| --- | --- | --- | --- | --- |
|  |  |  |  |  |
| ***All patients*** | 575 (100.0) | 409 (71.1) | 166 (28.9) |  |
|  |  |  |  |  |
| **Gender** |  |  |  | **0.106** |
| Female | 203 (35.0) | 136 (67.0) | 67 (33.0) |  |
| Male | 372 (65.0) | 273 (73.4) | 99 (26.7) |  |
|  |  |  |  |  |
| **Age** |  |  |  | **0.410** |
| <= 30 | 131 (22.8) | 88 (67.2) | 43 (32.8) |  |
| 31-40 | 210 (36.5) | 151 (71.9) | 59 (28.1) |  |
| 41-50 | 138 (24.0) | 96 (69.6) | 42 (30.4) |  |
| > 50 | 96 (16.7) | 74 (77.1) | 22 (22.9) |  |
|  |  |  |  |  |
| Mean (SD) | 39.7 (11.3) | 40.1 (11.5) | 38.7 (10.9) | **0.203** |
|  |  |  |  |  |
| **Route of transmission** |  |  |  | **0.083** |
| Heterosexual | 310 (53.9) | 215 (69.4) | 95 (30.6) |  |
| MSM | 184 (32.0) | 142 (77.2) | 42 (22.8) |  |
| PWID | 28 (4.9) | 16 (57.1) | 12 (42.9) |  |
| Unknown/Other | 53 (9.2) | 36 (67.9) | 17 (32.1) |  |
|  |  |  |  |  |
| **Country of origin** |  |  |  | **<0.05** |
| Sweden | 199 (34.6) | 156 (78.4) | 43 (21.6) |  |
| SSA | 208 (36.2) | 136 (65.4) | 72 (34.6) |  |
| East | 91 (15.8) | 58 (63.7) | 33 (36.3) |  |
| Other | 73 (12.7) | 55 (75.3) | 18 (24.7) |  |
| Unknown | 4 (0.7) | 4 (100.0) | 0 (0.00) |  |
|  |  |  |  |  |
| **Country of transmission** |  |  |  | **<0.01** |
| Sweden | 193 (33.6) | 154 (79.8) | 39 (20.2) |  |
| SSA | 178 (31.0) | 113 (63.5) | 65 (36.5) |  |
| East | 106 (18.4) | 71 (67.0) | 35 (33.0) |  |
| Other | 62 (10.8) | 47 (75.8) | 15 (24.2) |  |
| Unknown | 36 (6.3) | 24 (66.7) | 12 (33.3) |  |
|  |  |  |  |  |
| **HIV stage** |  |  |  | **0.598** |
| Non-LP | 244 (42.4) | 176 (72.1) | 68 (27.9) |  |
| LPnAH | 113 (19.7) | 76 (67.3) | 37 (32.7) |  |
| LPAH | 218 (37.9) | 157 (72.0) | 61 (28.0) |  |
| **CD4+ T-cell count at diagnosis** |  |  |  |  |
| Mean (SD) | 317.8 (257.2) | 323.0 (264.5) | 304.6 (238.2) | **0.663** |
|  |  |  |  |  |
