# Supplementary material for: Symptomatic Patients without Epidemiological Indicators of HIV Have a High Risk of Missed Diagnosis: A Multi-Centre Cross Sectional Study
Source: PLoS One. 2016 Sep 7;11(9):e0162503. doi: 10.1371/journal.pone.0162503 (PMC5014346; doi:10.1371/journal.pone.0162503)
Supplement: S2 Table — MSM, men who have sex with men; PWID, people with injecting drug use; SSA, sub-Saharan Africa; ‘East’, Eastern Europe, Asia and the Pacific region; ‘Other’, Western Europe, North and Latin America, the Caribbean, North Africa, Israel and the Middle East. (DOCX) [file pone.0162503.s002.docx]

**S2 Table. Descriptive and bivariate analysis of explanatory variables for the initiator of/reason for HIV-testing.**

|  |  |  |  |  |  |  |  |
| --- | --- | --- | --- | --- | --- | --- | --- |
| **Characteristics** | **Total**  **(Col %)** | **Patient initiated**  **(Row %)** | **Physician initiated:**  **Symptoms**  **(Row %)** | **Screening in high prevalent group**  **(Row %)** | **Contact tracing**  **(Row %)** | **Mothers health/ Blood donation**  **(Row %)** | **P-value** |
|  |  |  |  |  |  |  |  |
| ***Total cohort*** | 402 | 102 (25.2) | 133 (32.8) | 93 (23.0) | 44 (10.9) | 30 (7.4) |  |
|  |  |  |  |  |  |  |  |
| **HIV stage** |  |  |  |  |  |  | **<0.001** |
| Non-LP | 174 (43.3) | 63 (36.2) | 34 (19.5) | 40 (23.0) | 25 (14.4) | 12 (6.9) |  |
| LPnAH | 75 (18.7) | 17 (22.7) | 11 (14.5) | 26 (34.7) | 12 (16.0) | 9 (12.0) |  |
| LPAH | 153 (38.1) | 22 (14.4) | 88 (57.5) | 27 (17.7) | 7 (4.6) | 9 (5.9) |  |
|  |  |  |  |  |  |  |  |
| **Gender** |  |  |  |  |  |  | **<0.001** |
| Female | 134 (33.3) | 19 (14.2) | 39 (29.1) | 44 (32.8) | 8 (6.0) | 24 (17.9) |  |
| Male | 268 (66.7) | 83 (31.0) | 94 (35.1) | 49 (18.3) | 36 (13.4) | 6 (2.2) |  |
|  |  |  |  |  |  |  |  |
| **Age** |  |  |  |  |  |  | **<0.001** |
| <= 30 | 85 (21.1) | 22 (25.6) | 13 (15.3) | 29 (34.1) | 6 (7.1) | 15 (17.7) |  |
| 31-40 | 149 (37.1) | 41 (27.5) | 41 (27.5) | 35 (23.4) | 20 (13.4) | 12 (8.0) |  |
| 41-50 | 95 (23.6) | 25 (36.3) | 38 (40.0) | 19 (20.0) | 11 (11.6) | 2 (2.1) |  |
| > 50 | 73 (18.2) | 14 (19.2) | 41 (56.2) | 10 (13.7) | 7 (9.6) | 1 (1.4) |  |
|  |  |  |  |  |  |  |  |
| Mean (SD) | 40.2 (11.5) | 39.2 (10.4) | 45.3 (12.3) | 36.9 (10.2) | 39.3 (10.1) | 31.7 (8.0) |  |
|  |  |  |  |  |  |  |  |
| **Route of transmission** |  |  |  |  |  |  | **<0.001** |
| Heterosexual | 210 (52.2) | 40 (19.1) | 72 (34.3) | 52 (24.8) | 21 (10.0) | 25 (11.9) |  |
| MSM | 140 (34.8) | 55 (39.3) | 49 (35.0) | 16 (11.4) | 19 (13.6) | 1 (0.7) |  |
| PWID | 16 (4.0) | 4 (25.0) | 4 (25.0) | 6 (37.5) | 2 (12.5) | 0 (0.0) |  |
| Blood | 6 (1.5) | 0 (0.0) | 3 (50.0) | 2 (33.3) | 0 (0.0) | 1 (16.7) |  |
| Unknown/Other | 30 (7.5) | 3 (10.0) | 5 (16.7) | 17 (56.7) | 2 (6.7) | 3 (10.0) |  |
|  |  |  |  |  |  |  |  |
| **Country of origin** |  |  |  |  |  |  | **<0.001** |
| Sweden | 155 (38.6) | 55 (35.5) | 60 (38.7) | 17 (11.0) | 21 (13.6) | 2 (1.3) |  |
| “East”^1^ | 55 (13.7) | 18 (32.7) | 18 (32.7) | 10 (18.2) | 4 (7.3) | 5 (9.1) |  |
| SSA^2^ | 134 (33.3) | 16 (11.9) | 31 (23.1) | 55 (41.0) | 11 (8.2) | 21 (15.7) |  |
| Other^3^ | 54 (13.4) | 13 (24.1) | 23 (42.6) | 10 (18.5) | 6 (11.1) | 2 (3.7) |  |
| Unknown | 4 (1.0) | 0 (0.0) | 1 (25.0) | 1 (25.0) | 2 (50.0) | 0 (0.0) |  |
|  |  |  |  |  |  |  |  |
| **Country of transmission** |  |  |  |  |  |  | **<0.001** |
| Sweden | 151 (37.6) | 44 (29.1) | 53 (35.1) | 18 (11.9) | 28 (18.5) | 8 (5.3) |  |
| “East”^1^ | 70 (17.4) | 24 (34.3) | 28 (40.0) | 11 (15.7) | 3 (4.3) | 4 (5.7) |  |
| SSA | 111 (27.6) | 10 (9.0) | 30 (27.0) | 47 (42.3) | 8 (7.2) | 16 (14.4) |  |
| Other^2^ | 46 (11.4) | 19 (41.3) | 14 (30.4) | 10 (21.7) | 1 (2.2) | 2 (4.4) |  |
| Unknown | 24 (6.0) | 5 (20.8) | 8 (33.3) | 7 (29.2) | 4 (16.7) | 0 (0.0) |  |
|  |  |  |  |  |  |  |  |
